# Biological Cardiovascular Age Derived from Coronary CTA Reports Using a Large Language Model: A Novel Predictor of Major Adverse Cardiovascular Events?

**DOI:** 10.3390/diagnostics16091298

**Published:** 2026-04-26

**Authors:** Gudrun M. Feuchtner, Yannick Scharll, Johannes Deeg, Valentin Bilgeri, Philipp Spitaler, Malik Galijasevic, Michael Swoboda, Leonhard Gruber, Gerlig Widmann, Pietro G. Lacaita

**Affiliations:** 1 Department of Radiology, Innsbruck Medical University, 6020 Innsbruck, Austria; gudrun.feuchtner@i-med.ac.at (G.M.F.); johannes.deeg@i-med.ac.at (J.D.); leonhard.gruber@i-med.ac.at (L.G.); gerlig.widmann@i-med.ac.at (G.W.); 2 Department of Internal Medicine III, Cardiology & Angiology, Innsbruck Medical University, 6020 Innsbruck, Austria; valentin.bilgeri@outlook.com (V.B.); philipp.spitaler@i-med.ac.at (P.S.)

**Keywords:** biological age, coronary CTA, artificial intelligence, large language model, MACE, CAD-RADS, cardiovascular risk prediction, coronary artery disease

## Abstract

**Background/Objectives**: Coronary artery disease (CAD) remains the leading cause of death worldwide. Traditional cardiovascular risk assessment is based on chronological age and other clinical factors, with inherent limitations and poor accuracy. Objective was to estimate the artificial intelligence (AI)-enhanced biological cardiovascular age calculation derived from coronary computed tomography angiography (CTA) reports using a large language model (LLM), in predicting major adverse cardiovascular events (MACE). **Methods**: Coronary CTA reports were analyzed using a LLM (ChatGPT-4.0v, OpenAI), from symptomatic patients with suspected CAD who underwent coronary CTA for clinical indications. Patients in which the LLM successfully analyzed the key metrics (1) coronary artery calcium (CAC) score and (2) coronary CTA reports (coronary stenosis severity (CAD-RADS), high-risk anatomy, non-calcified plaque, cardiac function (LVEF and others) were included. **Results**: 386 CTA reports were uploaded, and 346 (89.6%) included. The mean biological age (bioAGE) was 57.2 ± 10.9 and the chronological 58.5 ± 10.8 years. 137 (39.6%) were women. The intra-individual deviation in bioAGE was high (median: 8.8; IQR 9.98). BioAGE exceeded chronological age in 45.4% patient and was lower or equal in 54.6%) MACE rate was 8.7% comprising 2 deaths, 5 myocardial infarctions, and 22 late revascularizations. The accuracy for prediction of MACE was higher for bioAGE (c = 0.768; 95% CI: 0.681–0.855, *p* < 0.001) compared to chronological age (c = 0.590; 95% CI: 0.492–0.689, *p* = 0.102) **Conclusions**: Biological age calculation from coronary CTA reports using LLM is feasible, yet intra-individual deviations are high. The accuracy for prediction of MACE is improved by bioAGE compared to chronological.

## 1. Introduction

Coronary artery disease (CAD) remains the leading cause of death worldwide [1]. Chronological age is a major factor in traditional cardiovascular disease risk assessment. However, conventional risk assessment based on age and cardiovascular risk factors (CVRF) has a poor accuracy (c = 0.5–0.6) [2] and a weak discriminatory power.

Aging is a heterogeneous process, and chronological age alone does not capture inter-individual variability in cardiovascular health. Biological age, by contrast, integrates physiological parameters reflecting more nuanced organ-specific damage and aging, potentially offering more accurate and personalized risk estimate, and heart age has been proposed as promising tool during the last years [3,4] but lacks more specific investigations. Biological age can be defined by a wide variety of measures ranging from organ specific functional parameters (liver enzymes, lipid panels or other blood samples [5], cardiac or lung function) to direct measures such as genetic profiling, DNA methylation and telomere length.

Cardiovascular imaging modalities such as coronary computed tomography angiography (CTA) have opened new horizons for assessing biological cardiovascular age. The coronary artery calcium (CAC) score—a cornerstone for risk assessment in asymptomatic individuals [6,7]—allows for an estimation of the “coronary age” by quantification of the coronary artery calcium (CAC) score [7]. Further, coronary CTA enables ultrahigh resolution imaging of vessel walls and an even a more nuanced evaluation of atherosclerosis, by assessing stenosis severity [8,9], quantifying plaque morphology and high-risk plaque (HRP) features [10,11], which have shown incremental value over traditional risk factors for risk assessment. In addition, cardiac function parameters such as left ventricular ejection fraction, stroke volume and cardiac output are derived from coronary CTA, adding further key metrics for cardiovascular health and completing a comprehensive framework for biological cardiovascular age calculation. Therefore, these parameters may allow for a more accurate biological cardiovascular age (BioAGE) estimation from coronary CTA reports for prediction of MACE than calendric chronological age.

The integration of artificial intelligence (AI), and specifically large language models (LLMs), represents a paradigm shift in the use of medical data. LLMs can parse unstructured text, interpret context, and extract relevant metrics for downstream analysis. In the context of coronary CTA, LLMs offer a unique capability to transform descriptive radiology reports into structured data, enabling the automated calculation of biological cardiovascular age [12].

Our study investigates the feasibility of calculating biological cardiovascular age (BioAGE) from coronary CTA reports, and evaluates whether the biological cardiovascular age, calculated from CTA reports using an LLM (ChatGPT-4.0v), is superior to chronological age in predicting major adverse cardiovascular events (MACE) in a retrospective observational study cohort of symptomatic patients referred to coronary CTA for clinical indications.

## 2. Material and Methods

### 2.1. Study Population

Inclusion criteria: Symptomatic patients with suspected CAD who underwent coronary CTA at our institution for clinical indications [13] were enrolled into our retrospective observational cohort study database (CONFIRM2 registry) [14].

Institutional review board (IRB) approval was obtained. Patients with known coronary artery disease (prior PCI, CABG, prior MI) or those with any other structural heart disease such as severe aortic stenosis referred for TAVI planning, were excluded from the database. The detailed criteria for patient recruitment have been described [14].

### 2.2. Coronary Computed Tomography Examination

Coronary CTA examinations were performed on a 128-slice dual-source CT scanner (Somatom *Definition FLASH*, *Siemens or DRIVE*) using 2 standardized acquisition protocols.

Coronary Artery Calcium (CAC) score. A non-contrast ECG-gated scan with standardized scan parameters (detector collimation 2 × 64 × 0.6 mm; 120 kV; image reconstruction 3 mm slice width, increment 1.5), and prospective ECG-triggering in dual source high –pitch mode was performed. The CAC score in Agatston Units (AU) [15] of all coronary arteries was calculated with automated software (Cardiac CT, SyngoVIA, Siemens Healthineers, Erlangen, Germany).

Coronary Computed Tomography Angiography (CTA) was appended with detector collimation of 2 × 64 × 0.6 mm and a z-flying spot and a rotation time 0.28 s. Prospective ECG-triggering was used in regular heart rates < 65 bpm (70% of RR-interval) and retrospective ECG-gating in heart rates > 65 bpm and irregular rates. An iodine contrast agent (Iopromide, Ultravist 370™, Bayer Vital GmbH, Leverkusen, Germany) was injected intravenously (flow rate 4–6 mL/s + 40 cc saline), triggered into the arterial phase (bolus tracking; 100 HU threshold; ascending aorta). Contrast volume ranged from 65 up to 120 mL. Axial images were reconstructed with 0.75 mm slice width (increment 0.4/medium-smooth kernel B26f) during the best diastolic and systolic phase. Curved multiplanar reformations (cMPR) and oblique interactive MPR using client-server based 3-D post-processing software (SyngoVia^TM^, Siemens Healthineers) were generated and the following CTA features were evaluated:

Coronary stenosis severity was scored visually according to CAD-RADS^TM^ [8] (0–5) as minimal (1) <25%, mild (2) 25–49.9%, moderate (3) 50–69.9%, severe (4) ≥70–99% and (5) occluded 100% [7] assisted by quantitative stenosis measurement using curved multiplanar reformations (cMPR) per coronary vessel and segment. Plaque morphology was described as non-calcified plaque (NCP), calcified or mixed plaque visually based on the CT attenuation. NCP was defined as hypoattenuating to the vessel lumen, and calcified plaque as hyperattenuating.

High risk plaque features (HRP) were quantified and defined as those with a low attenuating plaque component of less than 30 HU [10] or less than 60 HU and positive remodeling (±other high risk features such as spotty calcification or the napkin ring sign).

Left ventricular (LV) function parameters were calculated using automated contour tracking and LV volume segmentation over the entire cardiac cycle (SyngoVIA, Siemens Healthineers). LV ejection fraction (LVEF), enddiastolic and endsystolic volume, stroke volume and cardiac output were calculated and included in the report.

The structured standardized radiological CTA reports were extracted from our electronic health record system. Each CTA report included the following 3 key metrics: Coronary artery calcium (CAC) score, as Agatston Units (AU) [15], Coronary stenosis severity (CAD-RADS 2.0) classification, high-risk features and cardiac left ventricular function parameters: left ventricular ejection fraction (LVEF), end-diastolic volume (EDV), end-systolic volume (ESV), stoke volume (SV) and cardiac output.

Additional imaging-based indicators of myocardial or vascular aging, when reported such as severe aortic ectasia, or other abnormalities were included.

LLM-Based Estimation of Cardiovascular Biological Age from Coronary CTA reports: Structured standardized clinical radiological CTA reports were extracted from our electronic health record system. Only coronary CTA reports authored or co-signed by a SCCT Level III–equivalent certified readers with a high level of experience (>10 years of cardiac CT) were included in the LLM-based subanalysis. All CTA reports were from one site only, despite all CTA reports being in German language. The model processed reports in their original German language without prior translation.

Exclusion criteria for LLM subanalysis were ambiguous or inconsistent language, such as the absence of CAD-RADS classification or equivocal descriptors and non-conformal phrasing.

The eligible clinical coronary CTA reports were anonymized, saved as word.doc and uploaded to a large language model (LLM) (ChatGPT-4o, OpenAI, 2024, Version April–May? Temperature?). Semi-quantitative estimation of biological cardiovascular age from standardized coronary CTA reports was performed by providing the model with a structured prompt as follows #“*Please calculate the biological age from this clinical coronary CTA report*”.

The scripts with the results of the LLM query were supervised by a senior reader, a cardiovascular radiologist with >3 years of experience (EACVI certified) to ensure accuracy, plausibility and coherence.

If within the LLM-scripts one of the 3 coronary CTA report key metrics (CAC, CADRADS, Cardiac function) was missing or ambiguously stated, the prompt was adapted to specify missing metrics (e.g., “please also include CAC score” or “please include coronary stenosis severity (CAD-RADS)”), and the LLM-query was repeated.

The model synthesized the biological cardiovascular age (BioAGE) based on a clinical heuristic framework, by drawing a mean pooled estimate from the following key components:

- Coronary Artery Calcium (CAC) Score, weighted using the approximation: Biological Age = 39 + (CAC × 0.1)
- Coronary Stenosis severity and extent, following CAD-RADS classification:
  ○ CAD-RADS 2 → +5 years
  ○ CAD-RADS 3 → +10 years
  ○ CAD-RADS 4a–4b → +15–20 years
  ○ Multivessel disease (LAD, RCA, CX > 50%) → +15 years
  ○ High risk anatomy: LAD proximal segment >70% −> + 10 years
  ○ RCA or CX soft plaque only → +5 years
- Cardiac Functional markers such as:
  ○ LVEF < 50% or elevated EDV/ESV → +10 years
- Additional extracoronary findings considered to contribute to accelerated cardiovascular aging included, for example ascending aortic aneurysm or ectasia were included.

Supplement Figure S1 shows the results of a typical LLM query.

Finally, all biological cardiovascular age estimations were supervised by a senior reader, a cardiovascular radiologist with >3 years of experience (EACVI or ESCR certified for cardiovascular) and a supervisor with >10 years of experience to ensure accuracy, plausibility and coherence.

The data series was split into 4 groups of approximately 100 subjects (according to their consecutive numeric codes from the database), and the groups were analyzed separately with the LLM on different days with a time interval of >48 h.

The LMM performed both structured data extraction and contributed to calculation logic.

Cardiovascular outcome measures: Primary endpoint were major adverse cardiovascular events (MACE), defined as death, myocardial infarction according to standardized definitions [16] and late coronary revascularizations according to the CONFIRM2 [14] study protocol: Right censoring was performed. Follow-up data were collected at a pre-defined time point (after a minimum of 2–3 years up to 5–6 years). After an event occurred, the patient was censored.

Statistical analysis was performed using SSPS™ software (V21.0, SPSS Inc., Chicago, IL, USA) and MedCalc (V12.5, Ostend, Belgium). Quantitative variables are expressed as means ± standard deviation (SD), and categorical variables are expressed as absolute values and percentages. A *p*-value of <0.05 was considered as significant. Normal distribution of data was tested with Shapiro-Wilk test and histogram. Differences between parametric data were tested with the independent samples *t*-test for normally distributed and Mann-Whitey U for non-normally distributed data, and with the Chi-Square, or Fisher’s exact test for categorical data. Receiver operating curves (ROC) with area under the curves (AUC) were generated for the prediction of MACE by biological age and chronological age.

## 3. Results

**Figure 1:**
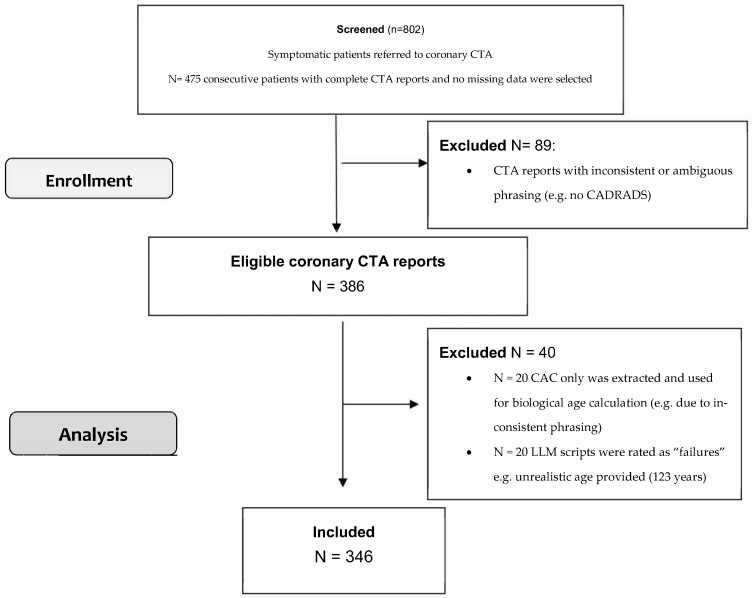
Flow chart.

Of 802 patients enrolled into our retrospective database, 475 patients with complete CTA reports and without missing data were included. and utilized for the CTA report analysis subproject. A total of 89 CT reports were excluded due to inconsistent language phrasing or ambiguous CTA reporting style (e.g., no CAD-RADS), or due to low-report quality from a non-certified low-level of experience radiologist reader with <3 years of experience. Figure 1 shows the flowchart of database screening, patient enrollment and coronary CTA report analysis. Finally, the clinical CTA reports of 386 patients were uploaded to the LLM.

**Figure 2:**
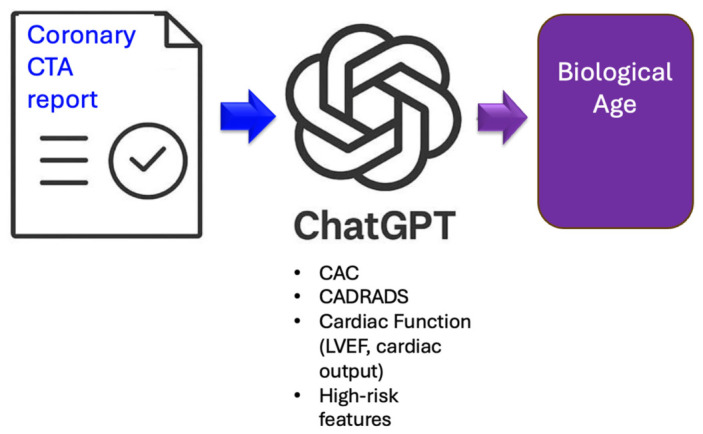
Data processing workflow: Coronary CTA report upload to the large language model (LLM) (Chat GPT 4o) and biological age (BioAGE) calculation from coronary CTA metrics.

Figure 2 illustrates the data processing workflow.

A total of 40 LLM scripts from CTA reports were rated as “failures” and were excluded. 20 scripts were excluded because an unrealistic biological age was provided (e.g., too high or too low age, e.g. 123 years or 17 years). In 20 coronary CTA reports, CAC only was extracted, while coronary stenosis could not be successfully added—despite repeating the query with specification. Reasons were for example ambiguous German language phrasing (e.g., using the synonymous word “Einengung” instead of “stenosis”, among others). A total of 346 patients were finally included in the analysis, in which both CAC and coronary CTA findings were included for biological age estimation. The success rate was 89.6%. Table 1 shows the study cohort profile and CTA parameters. The mean biological age was 57.2 ± 10.9 years (range, 39–95) (95% Confidence Interval: 56.03–58.36) and the chronological age 58.5 ± 10.8 years (range, 25–81) (95% CI 57.46–59.61).

**Figure 3:**
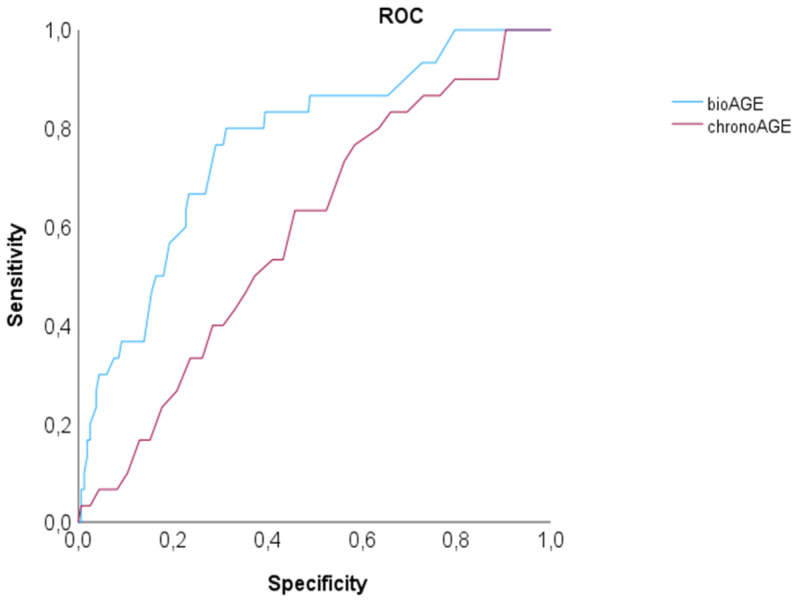
Receiver operating curve (ROC) for prediction of MACE by biological (BioAGE) compared to chronological age (chronoAGE) from coronary CTA reports: The AUC was higher for BioAGE compared to chronoAGE.

The mean deviation between biological and chronological age compared intra-individually was 10.48 ± 7.61 SD years (median: 8.8; IQR 9.98) (95% Confidence Intervall: 9.6–11.23). Biological age based on CTA reports was higher in 156 patients (45.4%) than the chronological, and lower in 189 (54.6%) (out of those equal in 3 of 189). MACE occurred in 30 of 346 patients (8.7%) (2 deaths (1 cardiac, 1 non-cardiac), 5 myocardial infarctions (4 NSTEMI + 1 STEMI) and 22 late revascularizations). On ROC, the AUC for prediction of MACE by biological age was higher compared to the chronological with c = 0.768 (95% CI: 0.681–0.855), *p* < 0.001 vs. c = 0.590 (95% CI: 0.492–0.689), *p* = 0.102 (Figure 3). The mean follow-up period was 4.2 years ± 2.8 years (median 4, IQR 1).

**Figure 4:**
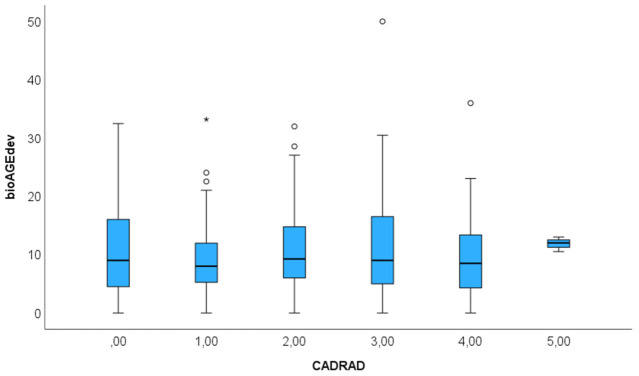
Boxplot: Biological cardiovascular age (BioAGE) deviation from coronary CTA reports among coronary stenosis severity (CADRADS) categories: the highest range of deviation was observed for CADRADS 0. (both negative and positive deviation values are displayed as positive values). * = outlier.

Sex-specific differences: 137 (39.6%) were women. Biological age deviation did not differ in men vs. women with 10.7 ± 7.8 vs. 10.07 ± 7.2 (Mann-Whitey U: *p* = 0.953). The BioAGE deviations among CADRADS categories were different, fueled by other features such as CAC score, high-risk anatomy, non-calcified plaque or left ventricular function (Figure 4).

Table 2 shows the consistency analysis of the ROC analysis for prediction of MACE by the LLM-queries. The dataset was split into 4 batches: 1st batch: (n = 94 LLM- scripts of the coronary CTA reports were included), 2nd batch (n = 149), 3rd batch (n = 252), 4th batch (final results n = 346). The AUC values for BioAGE and chronological age were not different for each series. Consistently, higher AUC values were observed for biological (BioAGE) compared to chronological age (ChronoAGE).

**Figure 5:**
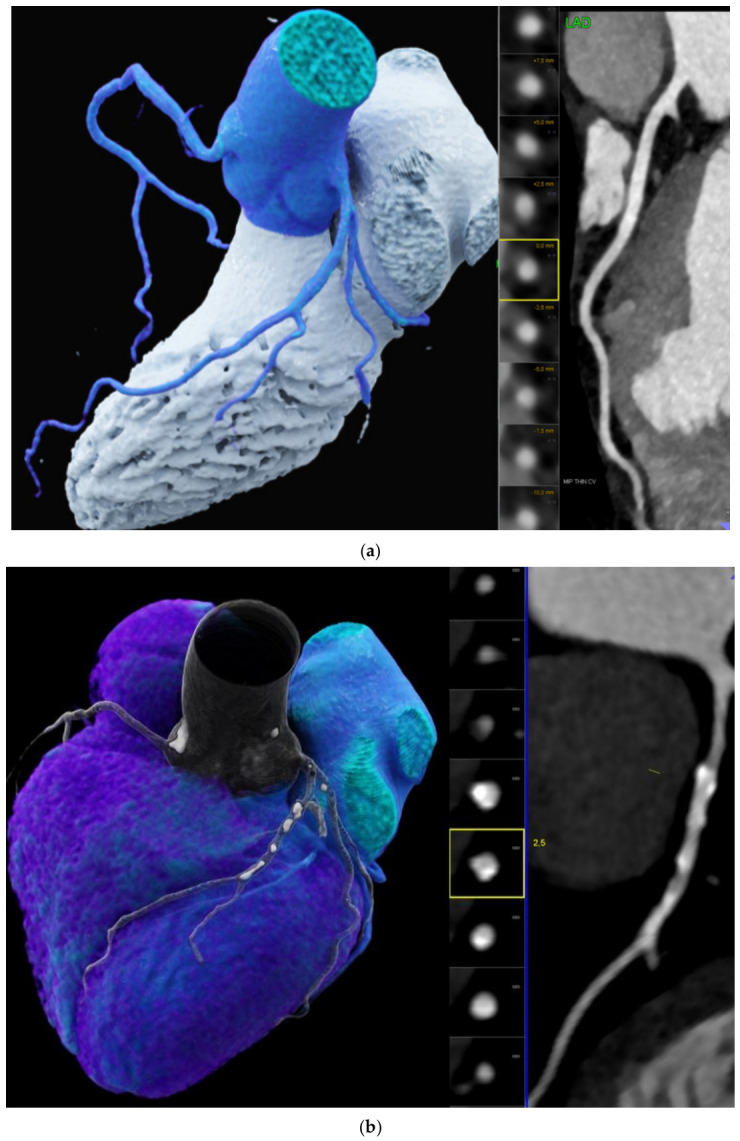
Coronary CTA images: Illustration of the CTA metrics synthesized for the standardized clinical CTA reports. (**a**) no coronary heart disease: in a patient with no coronary calcifications (CAC 0), no stenosis (CADRADS 0) and high LVEF 77%. His estimated biological age was 42.5 years (minus—19.5 years BioAge deviation) obtained from LLM analysis of his coronary CTA report. (**b**) Coronary heart disease in another patient with moderate coronary calcifications (CAC 215.3 AU) and moderate stenosis severity (CADRADS 3, 50–69%). His biological cardiovascular age (BioAGE) was 71 years (+5 years) based on the LLM analysis of CTA report metrics.

Figure 5 illustrates the coronary CTA imaging findings used for standardized clinical coronary CTA report generation for (a.) normal healthy heart and (b.) an aged heart with a high coronary calcification load and a higher biological age compared to chronological.

## 4. Discussion

Our study shows the feasibility of estimating biological cardiovascular age (BioAGE) from standardized clinical radiological coronary CTA reports using a LLM (Chat GPT-4ov, OpenAI) and demonstrates a superior accuracy for predicting MACE compared to chronological age. Of note, despite all coronary CTA reports were in German language, and success rate was 89.6%, underscoring the generalizability of LLM even when not in english language text.

The LLM interpreted diverse and unstructured radiological language in the majority of coronary CTA reports—as previously demonstrated [12,17]. Biological age was synthesized by key imaging features (CAC, coronary CTA features such as stenosis severity (CAD-RADS), other high-risk features such as high-risk anatomy (>70% proximal stenosis), or multivessel disease and functional parameters such as LVEF, cardiac output), which are known predictors of mortality [8,11]. The CONFIRM registry showed in 3547 patients during 5.4 years of follow-up, that MACE risk was higher for the atherosclerotic extent and number of segments involved [8] defined by coronary CTA. Further, both all-cause mortality and MACE increase along with coronary stenosis severity (CADRADS) [18]. Also, high-risk anatomy such as proximal coronary segment involvement increased risk in a large cohort (4644 patients) [19].

Interestingly, while the overall mean biological and chronological were similar in our cohort, the intra-individual discrepancies were high, with both negative and positive deviations at a high range. This highlights the potential of biological age as more sensitive personalized markers for cardiovascular health and longevity compared to conventional traditional measures such as chronological age or other cardiovascular risk factors.

Second, and most importantly, the biological age metric demonstrated comparable predictive accuracy with higher discriminatory power for MACE (c = 0.759) in our cohort compared to chronological age (c = 0.568), reinforcing its clinical utility as a prognostic biomarker. Our findings align with prior research emphasizing the prognostic significance of coronary plaque burden and left ventricular function. Studies have consistently shown that high-risk CT features such as increasing stenosis severity [18], extent of vessel disease [8] and higher total atherosclerotic plaque volume [20] and high-risk anatomy [19], as well as impaired myocardial function predict adverse cardiovascular outcomes independent of traditional risk factors. Accordingly, the combination of all CTA features has the capability of superior discriminatory power for cardiovascular risk stratification, by offering a more precise and personalized approach. Personalized risk stratification, with the integration of CTA imaging-based biomarkers such as CAC or CADRADS, has shown higher prognostic value than [21] conventional scores such as the Diamond Forrester, or the 4 major cardiovascular risk factors or ASCVD. Imaging captures more precisely the heterogenous process of aging, involving a variety of compartments on a cellular level, such as extracellular matrix being degraded by MMP-9 [22]. This has important clinical implications, especially is settings where therapeutic decisions regarding statin therapy are debated, and a reclassification of the individual risk has inherent consequences on patient management [23]. The PROMISE trial [11] has shown that high-risk plaque features from coronary CTA reclassify patients, in particular in low-risk individuals.

By leveraging LLMs for biological age estimation, we introduce a scalable, non-invasive, and interpretable tool. The biological age concept may resonate more effectively with patients and may enhance motivation for adhering to therapy (e.g., statin prescription) and other primary preventive interventions, such as behavioral and lifestyle modification (exercise, smoking cessation, dietary) [23] according to guidelines. The rate of adherence to statin therapy for prevention of cardiovascular disease is moderate; 25–50% of patients discontinue therapy within the first year, and the consistency of statin use decreases over time [24]. Statins have proven beneficial effects of coronary plaque morphology, by increasing the density of plaques the risk of rupture and acute coronary events decreases [25,26]. Adding the biological age (bioAGE) as communication tool may enhance the patient’s motivation to follow advice given for prevention of cardiovascular disease. For example, both exercise [27] and the Mediterranean diet [28] including olive oil decrease markers of inflammation triggering the aging process. Mounting scientific evidence prove that the speed of aging is vastly influenced by lifestyle and behavior of populations, and influences outcomes and prevalence in all age-related disease (cardiac and others such as neurodegenerative). Recent research on AI-based cardiovascular risk prediction supports the use of deep learning and natural language processing for automated phenotype generation and outcome prediction [29]. Both convolutional neural networks (CNNs) and transformer-based LLMs have shown high accuracy in extracting language from structured reports [12,17] and interpreting imaging biomarkers. When applied to coronary CTA, these models facilitate rapid, reproducible assessments that may outperform manual analysis. While prior AI tools—such as convolutional neural networks (CNNs) applied to raw CTA images—have shown promise in outcome prediction, their outputs often lack transparency. In contrast, our LLM-based method uses standard narrative reports and mimics clinical reasoning, offering both interpretability and real-world applicability. Biological age, as a novel marker of cardiovascular health based on emerging imaging-based metrics offers a multidimensional view of cardiovascular aging, by merging multiple established and novel coronary CTA imaging features such as CAC, stenosis severity, high-risk plaque and other functional markers into one metric. Therefore, it offers the unique advantage of a potential superior precision for prognostication of MACE. Importantly, the LLM approach has practical advantages—which is a major strength. It requires no additional imaging, or laboratory tests and leverages existing clinical data derived from coronary CTA reports. Its application can be extended across diverse healthcare settings globally, including low-resource environments, where complex decision-support systems are not available.

Study limitations. First, the retrospective design introduces inherent selection biases. Further, discrepancies in language and terminology across readers and institutions may affect generalizability. Third, only one LLM type (ChatGPT) has been tested, due to its most widespread use—but no comparison with other models such as Gemini or Claude was performed. Further, LLM results rates as “failures” were excluded (unrealistic age value), however the rate was low with 4.2%. Additionally, as the study is based on a single-center dataset with a modest number of events, and with reports in one language and a standardized reporting style, external generalizability may be limited. Future validation in multi-center and multi-language datasets would be valuable to confirm the robustness of the approach.

Finally, biological age is currently not used in traditional clinical practice due to lack of therapeutic management decisions. There is lack of scientific evidence how to integrate biological age into patient care and treatment plans, and further systematic research would be required to define the role of biological age.

## 5. Conclusions

Our study shows that biological cardiovascular age estimation from coronary CTA reports represents a novel biomarker with a comparable predictive performance with higher discriminatory value (AUC) in predicting major adverse cardiovascular events compared to chronological age. Biological age provides a personalized, interpretable, and easily communicable measure of cardiovascular health, biological age could foster greater patient engagement and adherence to primary preventive cardiovascular strategies promoting healthy aging and longevity.

Future outlook. The addition of biological age to current risk stratification tools, such as the pooled cohort equations (ACVD [30] or AHA prevent), may further improve cardiovascular risk prediction across populations.

## Figures and Tables

**Table 1:** Study cohort (n = 346).

**Age** (years)	58.5 ± 10.3
Women	137 (39.6%)
BMI kg/m^2^	26.3 ± 4.67
CVRF	
Smoking	94 (27.85%)
Arterial hypertension	146 (42.1%)
Positive family history	176 (52.2%)
Dyslipidemia	178 (51.4%)
Diabetes	26 (7.5%)
Coronary CTA report key metrics	
CAC	Mean 108.6 ± 263 (range, 0–24,328)
CADRADS	Mean 1.95 ± 1.34
CADRADS	
0	70 (20.2%)
1	55 (15.9%)
2	108 (31.2%)
3	58 (16.7%)
4 + 5	55 (15.9%)
**LVEF (%)**	69.7 ± 9.7 (range, 23–86)
**HRP**	88 (25.4%)

Abbreviations. CAC = coronary artery calcium score. LVEF = left ventricular ejection fraction. HRP = high—risk plaque. BMI = body mass index. Coronary stenosis severity classification (CADRADS). CTA = computed tomography angiography.

**Table 2:** Consistency analysis of the LLM-queries (split into 4 batches). 1st batch: (n = 94 LLM scripts included), 2nd batch (n = 149), 3rd batch (n = 252), 4th batch (final results n = 346) and not different for each batch. Consistently, higher AUC values were observed for biological (BioAGE) compared to chronological age (ChronoAGE). N = counts.

		BioAGE			ChronoAGE			MACE Rate
	N	AUC	95% CI		AUC	95% CI		
1st	94	0.797	0.505 –1.000	*p* = 0.026	0.570	0.393–0.743	*p* = 0.601	5 (5.3%)
2nd	149	0.759	0.591–0.928	*p* = 0.003	0.568	0.424–0.712	*p* = 0.433	17 (11.4%)
3rd	252	0.759	0.649–0.869	*p* < 0.001	0.562	0.058–0.327	*p* = 0.058	23 (9.1%)
4th batch	346	0.768	0.681–0.855	*p* < 0.001	0.590	0.492–0.689	*p* = 0.102	30 (8.7%)

## Data Availability

Data will not be shared public or in an open repository.

## Supplementary Materials

The following supporting information can be downloaded at: https://www.mdpi.com/article/10.3390/diagnostics16091298/s1, Figure S1: Case example; Figure S2: Prompt: #please specify how biological cardiovascular age was estimated?

## Abbreviations

The following abbreviations are used in this manuscript:

CAD	Coronary artery disease
CTA	Computed tomography angiography
CAC	Coronary artery calcium
CAD-RADS	Coronary Artery Disease Reporting and Data System
LVEF	Left ventricular ejection fraction
MACE	Major adverse cardiovascular events
LLM	Large language model
BioAGE	biological age
