# Supplementary material for: Biological Cardiovascular Age Derived from Coronary CTA Reports Using a Large Language Model: A Novel Predictor of Major Adverse Cardiovascular Events?
Source: Diagnostics (Basel). 2026 Apr 26;16(9):1298. doi: 10.3390/diagnostics16091298 (PMC13163461; doi:10.3390/diagnostics16091298)
Supplement: Supplementary file 1 [file diagnostics-16-01298-s001.zip › diagnostics-4261156-supplementary.pdf]

Supplement Figure S1. Case example

📘 Based on Published Risk Models:

🔹 **1. CAC Score and MESA Calculator**

- **CAC (Coronary Artery Calcium) Score = 0** is *strongly protective* and associated with:
  - <1% annual risk of a cardiac event
  - CV age ~5–10 years younger than actual age (based on MESA data)
  - For example:
    - At age 50, average CAC is ~10–30
    - A score of 0 = in the **lowest risk decile**

✅ So in your case:
CAC = 0 → CV age is 5–10 years younger, depending on sex and risk factors.

🔹 **2. Coronary Plaque (non-calcified)**

- Even though CAC = 0, your CT shows:
  - **Non-calcified plaque** in **proximal LAD**, <50% stenosis
  - CAD-RADS 2 (Mild atherosclerosis)
  - This suggests **subclinical disease**, typically seen in people in their **40s–60s** depending on risk factors
  - This **slightly offsets** the benefit of a CAC = 0

📉 So we reduce the 5–10 year benefit to **~3–7 years younger**, conservatively.

🔹 **3. Left Ventricular Function**

- **EF = 63%** → normal systolic function
- **LV volumes and mass** → within normal
- **Stroke volume and cardiac output** → possibly elevated due to fitness or hyperdynamic state
- No hypertrophy or dysfunction

✅ These support a **"younger" functional heart age.**

📊 Approximate Biological CV Age Calculation (Summary):

Component	Value	Effect on CV Age
CAC Score	0	↓ CV Age by ~5–10 yrs
Non-calcified plaque	CAD-RADS 2 (mild)	↑ CV Age by ~2–3 yrs
LVEF and LV function	Normal	Neutral or ↓ CV Age
**Overall Net Effect**		**↓ CV Age by ~5 years**

Supplement Figure S2: prompt: *#please specify how biological cardiovascular age was estimated?*

Heuristic Formula for Biological Age Estimation from Coronary CTA

- **ChronAge = Chronological Age**
- **ΔBA = Adjustment to Biological Age**
- **Biological Age = BA + ΔBA**

🔢 Adjustment Criteria (ΔBA)

Imaging Finding	Criteria	ΔChronAge
**Calcium Score**	0 AU	±0 years (age baseline or younger)
	1–99 AU	+5 years
	100–399 AU	+10 years
	400–999 AU	+15 years
	≥1000 AU	+20 years
**CAD-RADS**	0–1	0 years
	2	+5 years
	3	+10 years
	4a	+15 years
	4b or 5	+20 years
**LVEF**	<55% or ↑EDV/ESV	+10 years
**Plaque Risk**	Non-calcified plaque in LAD or RCA	+5 years
	High-risk plaque (NCP + PR/LAP)	+5–10 years
**High risk anatomy**	Proximal LAD >70%	+10 years
**Multivessel Disease**	LAD + CX + RCA > 50%	+15 years
**Myocardial Bridging (isolated)**	No penalty	
**Other Cardiac Abnormalities**		
